# Epigenomics: Pioneering a New Frontier in Cancer Research

**DOI:** 10.4172/2153-0645.1000e109

**Published:** 2012-01-23

**Authors:** Yana Chervona, Max Costa, Wei Dai

**Affiliations:** Department of Environmental Medicine, New York University, Langone Medical Center, Tuxedo, NY 10987, USA

Cancer typically occurs as the consequence of mutations or deregulated expression of genes that control cell survival, proliferation and/or death. Given its remarkable intricacy and complexity, the concept of cancer as a disease that can be greatly impacted by alterations in epigenetic regulation (and thus gene expression) has gained considerable momentum within the scientific community. In the 1990s, one focal point of epigenetic cancer research was ascertaining the extent of DNA methylation abnormalities and defining how DNA methylation influences expression of oncogenes and tumor suppressor genes in transformed cells [1]. However, during the past decade, this focus has been significantly broadened by the upsurge of research identifying and studying the role of molecules that affect chromatin dynamics (i.e. global DNA methylation and post-translational modifications of histones) in cancer cells. This new wave of research has given way to an emerging view of what may now be called “the cancer epigenome,” which consists of heritable abnormalities that occur in the absence of DNA sequence alterations in the genome [2].

DNA methylation is probably the most widely studied and commonly understood epigenetic alteration. It is involved in the regulation of a wide range of cellular and molecular processes including chromatin structure and remodeling, X-chromosome inactivation, genomic imprinting, silencing of transposable elements, chromosome stability, and gene expression [3,4]. Global hypomethylation, as well as hypomethylation of transposable elements, is associated with genomic instability, while hypermethylation of the gene promoter region is associated with transcriptional repression [5,6]. Likewise, post-translational modifications of histone tails play a critical role in chromatin remodeling, nuclear architecture, and gene transcription [7,8]. The functional consequences of alterations in global DNA methylation and histone modification patterns are numerous, ranging from subtle changes in behaviors to major clinical manifestations, including cancer.

With the emergence of the “cancer epigenome”, a considerable amount of time and effort is being invested in the characterization of epigenetic markers, of which novel molecular targets can be identified and explored for cancer drug development. Presently, there are four FDA-approved drugs with an “epigenetic mode” of action in use within clinics: DNA methyltransferase (DNMT) inhibitors 5-azacytidine (Vidaza), decitabine (20-deoxy-5-azacytidine, Dacogen), histone deacetylase (HDAC) inhibitors suberoylanilide hydroxamic acid (SAHA, Zolinza), and romidepsin (Istodax). Numerous other DNMT and HDAC inhibitors are currently being developed and evaluated in preclinical studies, as well as in various stages of clinical trials [9]. Notably, 5-Azacytidine and decitabine have been successful in treating myelodysplastic syndrome and myeloid leukemias [10–12]. Moreover, SAHA and romidepsin are currently being used for the treatment of cutaneous T-cell lymphoma [13]. However, studies have shown that both of these established drug classes exhibit a considerable limitation in their specificity largely due to lack of understanding for their exact mechanisms of action. Recent studies have shown that HDAC inhibitors have a substrate spectrum that is broader than originally thought, and are capable of deacetylating numerous proteins that are not associated with epigenetic regulation [14]. In parallel, the induction of global DNA demethylation via inhibition of DNA methyltransferases displays broad effects in changing DNA methylation patterns without tumor-specificity, which is believed to promote, rather than suppress, oncogenic processes in many cancer patients [9,15]. Furthermore, drugs that perturb global DNA demethylation and/or normal histone modifications can potentially harm the function of adult stem cell populations in cancer patients [9]. Admittedly, the current repertoire of anti-cancer drugs that are based on perceived epigenetic targets has significant limitations in clinical practice.

It is well known that cancer cells exhibit aberrant DNA methylation patterns and specific changes in histone modifications, but the epigenetic alterations that may precede and/or contribute to the initiation and progression of the disease are poorly understood. Recent technological advances in high throughput DNA sequencing and epigenetic profiling has revolutionized our understanding of the development and progression of many vexing diseases, including cancer. With respect to epigenetic cancer therapy, a higher degree of specificity could be achieved if the drugs were directed against a cancer/tumor-specific epigenetic modification pattern. For example, it has been demonstrated that genes that are affected by *de novo* DNA methylation during carcinogenesis are pre-marked by histone H3 lysine 27 trimethylation (H3K27me3), suggesting that tumor-specific targeting of *de novo* methylation is pre-programmed by an established epigenetic system, which normally marks genes for repression [16]. In addition, H3K27 trimethylation is also part of “bivalent” chromatin domains, which consist of large regions of H3K27me3 and harbor smaller regions of histone H3 lysine 4 methylation (H3K4me3), a mark of gene expression activation. It has been proposed that bivalent domains silence developmental genes in embryonic stem cells, while keeping them poised for activation, and that the relationship between H3K27me3 and *de novo* DNA methylation may potentially reflect the presence of a stem cell-like epigenetic program in cancer cells [17,18]. Moreover, a number of mutations have been detected in genes associated with DNA methylation (e.g., TET2, IDH1/2, DNMT3A) and could potentially serve as patient stratification biomarkers for treatment with a demethylation drug [9]. Covalent chromatin modification patterns could also potentially serve as biomarkers of exposure to carcinogenic agents, and may provide further insight into tumor initiation and progression induced by those agents. In fact, changes in DNA methylation and global histone modifications have already been reported in human populations who are exposed to carcinogenic metals [19–21].

The role of epigenomics in cancer research is expanding in its scope and depth. FDA-approved cancer therapy drugs that primarily target DNA methylation and global histone modifications are being increasingly used in clinical practices, and many more leads are being developed and evaluated at the time of this writing. Genomic and epigenomic profiling of tumors, along with epigenetic biomarkers of exposure to carcinogenic agents, is on the rise due to the advent of new, powerful sequencing technologies and bioinformatics tools. It’s clear that cancer treatment approaches in the future will demand information of both genomic and epigenomic analysis of tumor cells. We anticipate exciting and novel discoveries in the epigenomic arena. However, the ultimate success of these endeavors rests on our ability to translate these discoveries into better diagnostic and treatment approaches for cancer.
